# Chromene-Containing Aromatic Sulfonamides with Carbonic Anhydrase Inhibitory Properties

**DOI:** 10.3390/ijms22105082

**Published:** 2021-05-11

**Authors:** Andrea Angeli, Victor Kartsev, Anthi Petrou, Mariana Pinteala, Volodymyr Brovarets, Sergii Slyvchuk, Stepan Pilyo, Athina Geronikaki, Claudiu T. Supuran

**Affiliations:** 1 Neuro Farba Department, Sezione di Scienze Farmaceutiche, Università degli Studi di Firenze, Via Ugo Schiff 6, Sesto Fiorentino, 50019 Florence, Italy; claudiu.supuran@unifi.it; 2 Centre of Advanced Research in Bionanoconjugates and Biopolymers, Petru Poni Institute of Macromolecular Chemistry, Aleea Grigore Ghica-Voda, No. 41A, 700487 Iasi, Romania; pinteala@icmpp.ro; 3 InterBioScreen, Chernogolovka 142432, Moscow Region, Russia; vkartsev@ibscreen.chg.ru; 4 Department of Pharmacy, School of Health, Aristotle University of Thessaloniki, 54124 Thessaloniki, Greece; anthi.petrou.thessaloniki1@gmail.com; 5 Department of Chemistry of Bioactive Nitrogen-Containing Heterocyclic Bases, V.P. Kukhar Institute of Bioorganic Chemistry and Petrochemistry, NAS of Ukraine 1, Murmanska St, 02094 Kyiv, Ukraine; brovarets@bpci.kiev.ua (V.B.); s.slivchyk@mail.enamine.net (S.S.); stepanpilyo@ukr.net (S.P.)

**Keywords:** carbonic anhydrase, inhibitors, metalloenzymes, Chromene

## Abstract

Carbonic anhydrases (CAs, EC 4.2.1.1) catalyze the essential reaction of CO_2_ hydration in all living organisms, being actively involved in the regulation of a plethora of patho/physiological conditions. A series of chromene-based sulfonamides were synthesized and tested as possible CA inhibitors. Their inhibitory activity was assessed against the cytosolic human isoforms hCA I, hCA II and the transmembrane hCA IX and XII. Several of the investigated derivatives showed interesting inhibition activity towards the tumor associate isoforms hCA IX and hCA XII. Furthermore, computational procedures were used to investigate the binding mode of this class of compounds, within the active site of hCA IX.

## 1. Introduction

The carbonic anhydrases (CA’s) are metalloenzymes that catalyze the formation of bicarbonate from carbon dioxide in two steps [1,2,3,4]. Sixteen isoforms have been known up to now in humans, five of which CAs are cytosolic (CA I–III, VII, and XIII), two are mitochondrial (CA VA and VB), one is secreted (CA VI), and the others are membrane-bound (CA IV, IX, XII, XIV and XV) [5]. Many CA subtypes are interesting targets for the design of pharmacological agents useful, for example, as antiglaucoma, anticonvulsant, antiurolithic, and for the treatment of obesity [6]. Isoforms CA IX and CA XII were discovered to be overexpressed and associated with many tumors, where they are involved in processes related with cancer progression [7,8,9]. Hence, small molecules targeting CA IX/XII inhibition may be an attractive strategy against cancer. However, due to high homology among the human isoforms, selectivity towards one of them is of the highest importance to prevent off-target related side effects. The classical CA inhibitors (CAIs) are the primary sulfonamides, which coordinate the zinc ion with their terminal deprotonated nitrogen atom and have been in clinical use for more than 70 years as diuretics and systemically acting antiglaucoma drugs [10]. Chromenes have attracted the interest of the scientific community due to their wide range of biological activities, such as antimicrobial [11,12], anticancer [13,14], anti-inflammatory [15,16], and CA inhibitors [17,18,19,20,21], nematicidal [22], antiallergic [23] Some sulfonamides also show antimicrobial [24,25], anticancer [26,27], antidiabetic [28], anti-inflammatory [15,29], and antitubercular [30] activities. Taking into account all of the above-mentioned, here we report the synthesis of (4-sulfamoylphenyl)-4*H*-chromene-2-carboxamide derivatives for its incorporation into one scaffold chromene and sulfonamide moieties. The experimental studies were developed together with in silico techniques, aimed to propose a reliable binding disposition for this class of CA Is.

## 2. Results

### 2.1. Chemistry

**Scheme 1:**
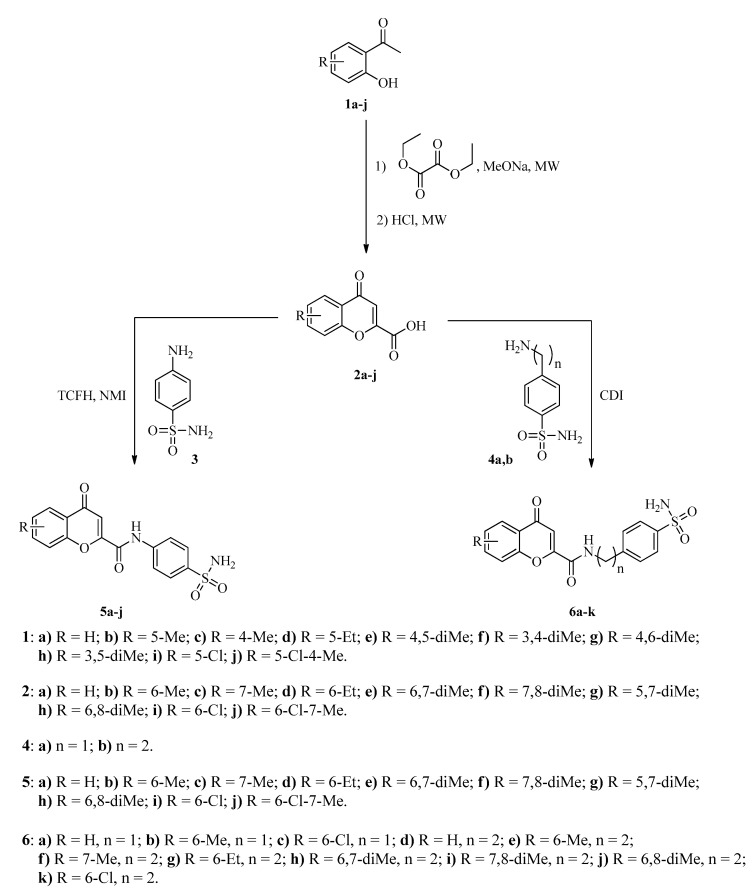
General synthetic strategy followed to obtain target compounds (**5a**–**j**) and (**6a**–**k**).

All the target compounds have been synthesized using the well-known strategy depicted in Scheme 1. It implies the synthesis of diverse 4-oxo-4*H*-chromene-2-carboxylic acids as well as amides mini array, which contains a streptocid moiety. The synthesis was started with commercially available 2′-hydroxyacetophenones **1a**–**j**, applying Fernando Cagide’s optimized synthetic route [31]. The desired carboxylic acids (**2a**–**j**) were synthesized using microwave irradiation in the presence of diethyl oxalate and sodium methoxide with very high yields (93–97%). To reach our final amides, we used two different methodologies that have a common characteristic—the formation of acyl imidazoliums. These highly reactive acyl transfer intermediates have been recognized as important species for amide bond formation.

As a part of the ongoing studies that are focusing on the synthesis of big libraries for biological screening, we discovered that the combination of N,N,N′,N′-tetramethyl­chloroformamidinium hexafluoro-phosphate (TCFH) and N-methylimidazole (NMI) is a mild method for in situ generation of highly reactive acyl imidazoliums, allowing for the formation of the amides in high yields. This approach is especially efficient for electron-deficient amines. Because of the low nucleophilicity of 4-aminobenzene-sulfonamide **3**, common coupling agents such as CDI, HATU, EDC, and EDCI with TEA or DIPEA, as a base in DMF, are not suitable for amide synthesis. Using the TCFH-NMI method, we have managed to access anilides **5a**–**j** in very good yields. In order to expand our screening library and to investigate an impact of aliphatic linker on bioactivity, we synthesized a set of amides based on 4-(aminomethyl)benzene­sulfonamide **4a** and 4-(2-aminoethyl)benzenesulfonamide **4b**. In this case, we can use the simple amide coupling agent CDI, considering the high nucleophilic properties of both amines. In this work, we synthesized two new 4-oxo-4*H*-chromene-2-carboxylic acids using modern microwave methodology, in which a set of amides have been subsequently produced.

### 2.2. Evaluation of CA Inhibitory Activity

All compounds were evaluated for their inhibitory activity against human CA isoforms, hCA I, hCA II, hCA IX, and hCA XII, and results are presented in Table 1. The result of biological evaluation revealed that all compounds showed activity against all isoforms, here tested, with different ranges of inhibition constants. The K_i_ values of compounds against hCA I ranged from 213.7 to 5314 nM. The best activity was achieved for compounds **6i** (K_i_ = 213.6 nM) and **6e** (K_i_ = 246.7 nM), higher than that of acetazolamide (AAZ) used as reference drug. On the other hand, the lowest activity was observed for compound **5b** with K_i_ = 5314 nM followed by compound **6j** (K_i_ = 4412 nM). The structure activity relationships (SAR) study revealed that, in general, derivatives of 4-oxo-N-(4-sulfamoylphenethyl)chroman-2-carboxamide are more potent hCA I inhibitors than 4-oxo-N-(4-sulfamoylphenyl)chroman-2-carboxamide and derivatives 4-oxo-N-(4-sulfamoylbenzyl)chroman-2-carboxamide. The presence of methyl groups in positions 7 and 8 of 4*H*-chromen-4-one moiety of -sulfamoylphenethyl derivatives is beneficial for hCA inhibitory activity, while the presence of 7-Me group at 4H-chromen-4-one moiety in case of sulfamoylphenyl derivatives was detrimental. As far as cytosolic hCA II isoform is concerned, the compounds showed, in general, better activity than against hCA I. Two compounds, **5f** and **6f,** exhibited excellent activity on isoform hCA II, demonstrating K_i_ values of 9.3 and 7.5 nM, higher than the reference drug AAZ (K_i_ = 12.1 nM). Compound **6d** with K_i_ value of 16.6 nM was comparable with the reference drug. The SAR revealed that the presence of a methyl group in position 7 of 4*H*-chromene-4-one, as well as CH_2_CH_2_ group between 4-oxo-4*H*-chromene-­2--carboxamide and benzensulfonamide (**6f**)**,** is beneficial for CA II inhibitory activity. Removal of a methyl group from position 7 of compound **6f** led to compound **6d** being twice less active than **6f**. On the other hand, the addition of one more methyl group in position 8 of the previous compound and removal of CH_2_CH_2_ group resulted in compound **5f**, with decreased activity compared to **6f**, but still more active than AAZ.

Removal of both methyl groups from compound **5f** led to compound **5a** (K_i_ = 35.1 nM), which is almost four times less active than compound **5f**, while removal of Me group from position 8 of compound **5f,** decreased more the activity **(**K_i_ = 39.5 nM). The presence of 6-Cl, 7-Me substitution on 4*H*-chromen-4-one moiety of sulfamoylphenyl derivatives was found to be very negative for hCA II inhibitory activity. In the case of hCA IX, compounds showed activity with K_i_ ranging from 16.6 to 3285 nM. Three compounds, 4-oxo-N-(4-sulfamoylphenyl)chroman-2-carboxamide **5a** (K_i_ = 16.6 nM); 7,8-dimethyl--4-oxo-N-(4-sulfamoylphenyl)chroman-2-carboxamide **5f** (K_i_ = 19.5 nM); and 5,8-dimethyl-4-oxo-N-(4-sulfamoylphenyl)chroman-2-carboxamide **5h** (K_i_ = 22.5 nM) displayed excellent activity superior compared to AAZ (K_i_ = 25.7 nM). 

The structure–activity relationships revealed that unsubstituted 4-oxo-N­-(4-sulfamoylphenyl)chroman-2-carboxamide (**5a**) was beneficial for hCA IX inhibition. The introduction of two methyl group at position 7 and 8 of the previous compound revealed a decrease in activity, leading to compound **5f** still being more active than AAZ. Shifting the 7-Me group to position 6 (**5h**) decreased the activity even more, but the compound remained still more active than AAZ. Introduction to unsubstituted compound (**5a**) chlorine at position 6 resulted in three times lesser active compound (**5i**). The presence of 6-Cl substituent at 4*H*-chromen-4-one moiety and methyl group between 4-oxo-4*H*-chromene-2-carboxamideand benzensulfonamide (**6e**) appeared to be detrimental for hCA IX inhibitory activity. For hCA XII, the inhibition constant ranged from 20.1 to 619.1 nM. The best activity against this isoform was exhibited by compounds **5a**, **6h**, and **5h** with K_i_ values of 20.1, 22.6, and 26.8 nM, respectively, compared to AAZ (K_i_ = 5.7 nM). None of compounds did not exert the activity of AAZ against hCA XII. Nevertheless, the unsubstituted 4-oxo-N-(4-sulfamoylphenyl)chroman-2-carboxamide was positive for hCA XII inhibitory activity, while the substitution by a 7-Me group of 4*H*-chromen-4-one moiety of -sulfamoylphenethyl derivatives is unfavorable for this kind of activity. Finally, it should be mentioned that compound **5a** was the most active one hCA IX with a selectivity index (SI) of 31.67 compared to hCA I, while compound **6f** was the most active hCA II with SI 44.47 compared to hCA I and 44.97 to hCA XII.

### 2.3. Molecular Modeling Studies

In order to explain the inhibition mechanism of the tested compounds, molecular docking studies were performed. For the in-silico experiments, ligands **5a**, **5h**, **6h**, **6f**, **6d**, and **6i** were selected as representatives of the whole set. Docking studies showed that all tested compounds bind in a deprotonated form, as anions (negative nitrogen of the sulfonamide group), chelating the Zn(II) ion of the active site of the enzymes [32]. It is known that all the active isoforms of human CAs have a similar active site architecture, consisting of three conserved His residues (His94, His96 and His119) acting as zinc ligands and another two conserved residues Thr199 and Glu105, acting as “gate keepers” [33,34,35,36]. Nevertheless, these isoforms differ in the amino acids mostly in the middle and to the exit of the active site cavity.

**Figure 1:**
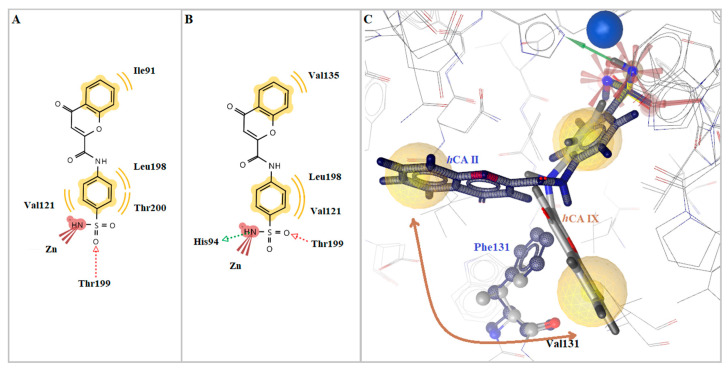
(**A**) 2D interaction diagram of compound 7 docking pose interactions with the key amino acids in hCA II, (**B**) in hCA IX, and (**C**) Superposition of compound 7 bound to hCA IX (grey) in comparison to hCA II (blue), with specific residues labeled. Active site zinc is shown as a blue sphere, red dotted and green arrows indicate H-bond, and yellow spheres indicate hydrophobic interactions. Brown double-headed arrow indicates the direction of conformational change of the compound bound to hCA IX- in comparison to hCA II.

According to docking studies, these differences in the active site of the enzymes are the reason for the selectivity of the compounds against each isoform. The N-C=O linker allow compounds to adopt several conformations and interactions within the enzyme active site. These conformations, depending on the nature of the amino acids of the active site cavity, can affect the inhibition profile of the ligands. For instance, compound **5a**, which has a K_i_ for hCA II of 35.1 nM and a lower K_i_ for hCA IX of 16.6 nM, adopts a much different conformation upon binding both hCAs. This is probably due to bulky hydrophobic residue Phe131 in hCA II enzyme, unlike that of the smaller residue Val131 in hCA IX, which allows ligands to freely enter the active site in a conformation that favors interactions with residues in the hydrophobic pocket and increases selectivity (Figure 1). The superposition of the two structures of hCAs bound to compound **5a**, revealed that this residue (Phe131) could indeed cause steric hindrance (Figure 1C). Therefore, **5a** adopts a different conformation within the active site of hCA II enzyme with less interactions and therefore less stability of the complex and probably explains the experimental lower K_i_ for hCA II. It is north worthy to mention that the sulfonamide is involved in H-bond formation with the backbone of Thr199 of both isoforms and in an additional H-bond formation with the backbone of His94, which further stabilizes the complex ligand hCA IX and contributes to the high inhibition potency of the compound (Figure 1A,B and Table 2).

**Figure 2:**
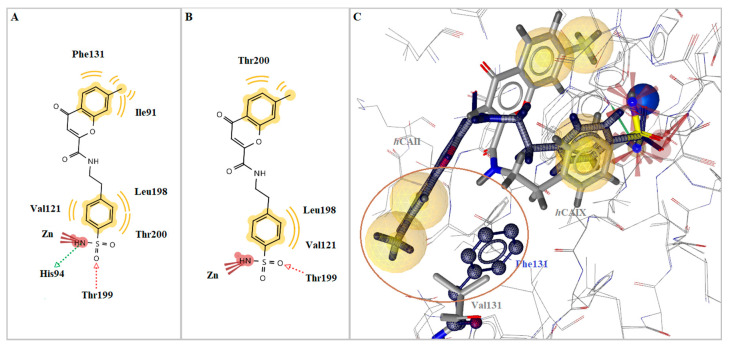
(**A**) 2D interaction diagram of compound 17 docking pose interactions with the key amino acids in hCA II, (**B**) in hCA IX, and (**C**) superposition of compound 17 bound to hCA IX (grey) in comparison to hCA II (blue), with specific residues labeled. Active site zinc is shown as a blue sphere, red dotted and green arrows indicate H-bond, and yellow spheres indicate hydrophobic interactions. Brown circle.

On the other hand, compounds **6h**, **6f**, **6d**, and **6i** differ from compounds **7** and **11** mainly by the presence of an ethylene linker between the benzimidazole scaffold and the side phenyl ring. This ethyl linker provides flexibility to the compounds and maximizes the favorable interactions in hCA IX and hCA XII, increasing the inhibition potency (compound **6h**) but, on the other hand, it also allows a better fit into the hCA I and hCA II isoform structures, affecting the ligand selectivity profile (compounds **6f**, **6d**, and **6i**). In the case of compound **6f**, the flexible ethyl linker leads the compound to adopt a fold conformation inside the larger active site cavity of hCA IX, unlike to the extended conformation of the hCA II isoform structure (Figure 2C). In both structures, negative nitrogen of the sulphonamide group chelates the Zn (II) ion and the interaction forms a H-bond with the side chain of conserved residue Thr199, but the extended conformation of the compound into the hCA II isoform structure allows the formation of hydrophobic interactions between the benzene ring of the compound and the residues Phe131 and Ile91 (Figure 2A,B and Table 2). These interactions further stabilize the complex ligand-enzyme and positively impacts the selectivity profile of the compound (K_i_ for hCA II of 7.5 nM and a lower K_i_ for hCA IX of 64.8 nM).

## 3. Materials and Methods

### 3.1. Chemistry

Reagents, chemicals, starting materials (**1a**–**j**, **3**, **4a**,**b**), and solvents were obtained from commercial sources and used without further purification. Melting points were determined on an Microquimica MQAPF-302 apparatus (Palhoça, Brazil). NMR spectra were recorded on a Avance III HD Bruker spectrometer (Rheinstetten, Germany). with chemical shifts values (*δ*) in ppm relative to TMS using the residual DMSO-d6 signal as an internal standard. High-resolution mass spectra (HRMS) were recorded on an LTQ Orbitrap Discovery mass spectrometer (Thermo Fisher Scientific, Bremen, Germany). This system combines an LTQ XL linear ion-trap mass spectrometer and an Orbitrap mass analyzer. The analyses were performed by direct infusion of the sample in DMSO (flow rate of 10 mL/min) in a positive-ion mode, using electrospray ionization (ESI). For the elemental composition, the calculations used the specific tool included in the Qual Browser module of Xcalibur (Thermo Fisher Scientific, release 2.0.7) software. Microwave-assisted synthesis was performed in a Biotage® Initiator Microwave Synthesizer (Biotage, Uppsala, Sweden). The progress of reactions was monitored by thin-layer chromatography (TLC) using precoated TLC sheets with Ultraviolet (UV) fluorescent silica gel (Merck, 60F254, Germany. HPLC (Fuji Silysia Chemical Ltd., Aichi-ken, Japan) purification method: mobile phase, system 20–70% 0–5min H_2_O/MeOH, flow: 30 mL/min (loading pump 4 mL/min methanol); column: Chromatorex 18 SMB100-5T 100 × 19 mm 5 μm.

#### 3.1.1. General Procedure for Synthesis of Compounds (**2a**–**j**)

The appropriate 2′-hydroxyacetophenone (**1a**–**j**) (1.16 mmol, 1 eq.) was dissolved in dioxane (2 mL) in a MW vial. Then diethyl oxalate (3.49 mmol, 474 µL, 3 eq.) and a solution of MeONa in MeOH (2.32 mmol, 531 µL, 25% *w*/*w*, 2 eq.) were added. The resulting solution was heated to 120 °C for 20 min. Then, a solution of HCl (18 mmol, 3 mL, 6 M) was added and the reaction was heated to 120 °C for 40 min. The reaction mixture was poured into water (50 mL) and the solid formed was filtered and washed with water. The solid was then dried, washed with dichloromethane, and dried again. Carboxylic acids **2a**–**e**, **h**–**j** had been synthesized previously by means of different methods and their QC data did not differ from ones we obtained [34,35,36,37].

#### 3.1.2. 7,8-Dimethyl-4-oxo-4*H*-chromene-2-carboxylic Acid (**2f**)

93% yield. MP = 259–261 °C. ^1^H NMR (400 MHz, DMSO-*d*_6_) *δ*: 14.20 (bs, 1H, COOH), 7.81 (d, *J* = 7.7 Hz, 1H, Ar), 7.42 (d, *J* = 7.6 Hz, 1H, Ar), 6.85 (s, 1H, Ar), 2.47 (s, 3H, CH_3_), 2.41 (s, 3H, CH_3_). MS (ESI): *m*/*z* (%) = 219.1 (13.2) [M + H]^+^. Anal. Calcd. for C_12_H_10_O_4_ (%): C, 66.05; H, 4.62. Found (%): C, 66.18; H, 4.59.

#### 3.1.3. 5,7-Dimethyl-4-oxo-4*H*-chromene-2-carboxylic Acid (**2g**)

97% yield. MP = 264–266 °C. ^1^H NMR (400 MHz, DMSO-*d*_6_) *δ*: 14.25 (bs, 1H, COOH), 7.61 (s, 1H, Ar), 7.38 (s, 1H, Ar), 6.87 (s, 1H, Ar), 2.55 (s, 3H, CH_3_), 2.39 (s, 3H, CH_3_). MS (ESI): *m*/*z* (%) = 219.2 (11.4) [M + H]^+^. Anal. Calcd. for C_12_H_10_O_4_ (%): C, 66.05; H, 4.62. Found (%): C, 66.09; H, 4.65.

#### 3.1.4. General Procedure for Synthesis of Compounds **5a**–**j**

To the 4-oxo-4*H*-chromene-2-carboxylic acid (**2a**–**j**) (1.52 mmol, 1.0 eq.), 4-aminobenzene-sulfonamide 3 (0.314 g, 1.82 mmol, 1.2 eq.) and *N*-methylimidazole (0.42 mL, 5.33 mmol, 3.5 eq.) combined and dissolved in MeCN (4 mL)) TCFH (0.517 g, 1.83 mmol, 1.2 eq.) was added in a single portion. The reaction was stirred until complete by LCMS (21 h). The reaction mixture was then diluted with ethyl acetate (6 mL) and water (4 mL). The layers were separated, the aqueous layer was extracted with ethyl acetate (4 mL), and the combined organics were washed with water (4 mL), dried with MgSO_4_, filtered, and concentrated before purification by HPLC to afford final amides with excellent yields.

#### 3.1.5. 4-Oxo-N-(4-sulfamoylphenyl)-4*H*-chromene-2-carboxamide (**5a**)

93% yield. MP = 296–298 °C. ^1^H NMR (400 MHz, DMSO-*d*_6_) *δ*: 10.92 (s, 1H, NH), 8.08 (dd, *J* = 7.8, 1.5 Hz, 1H, Ar), 7.98 (d, *J* = 8.7 Hz, 2H, Ar), 7.93–7.81 (m, 4H, Ar), 7.54 (t, *J* = 7.8 Hz, 1H, Ar), 7.23 (s, 2H, SO_2_NH_2_), 6.99 (s, 1H, Ar). MS (ESI): *m*/*z* (%) = 345.1 (17.6) [M + H]^+^. Anal. Calcd. for C_16_H_12_N_2_O_5_S (%): C, 55.81; H, 3.51; N, 8.14; S, 9.31. Found (%): C, 55.87; H, 3.49; N, 8.18; S, 9.28.

#### 3.1.6. 6-Methyl-4-oxo-N-(4-sulfamoylphenyl)-4*H*-chromene-2-carboxamide (**5b**)

89% yield. MP = 307–308 °C. ^1^H NMR (400 MHz, DMSO-*d*_6_) *δ*: 10.97 (s, 1H, NH), 8.02–7.86 (m, 5H, Ar), 7.74 (s, 2H, Ar), 7.34 (s, 2H, SO_2_NH_2_), 6.97 (s, 1H, Ar), 2.45 (s, 3H, CH_3_). MS (ESI): *m*/*z* (%) = 359.1 (20.3) [M + H]^+^. Anal. Calcd. for C_17_H_14_N_2_O_5_S (%): C, 56.98; H, 3.94; N, 7.82; S, 8.95. Found (%): C, 57.09; H, 3.99; N, 7.76; S, 9.08.

#### 3.1.7. 7-Methyl-4-oxo-N-(4-sulfamoylphenyl)-4*H*-chromene-2-carboxamide (**5c**)

91% yield. MP = 309–311 °C. ^1^H NMR (400 MHz, DMSO-*d*_6_) *δ*: 10.87 (s, 1H, NH), 7.99–7.84 (m, 5H, Ar), 7.61 (s, 1H, Ar), 7.34 (d, *J* = 8.4 Hz, 1H, Ar), 7.25 (s, 2H, SO_2_NH_2_), 6.95 (s, 1H, Ar), 2.53 (s, 3H, CH_3_). MS (ESI): *m*/*z* (%) = 359.1 (20.4) [M + H]^+^. Anal. Calcd. for C_17_H_14_N_2_O_5_S (%): C, 56.98; H, 3.94; N, 7.82; S, 8.95. Found (%): C, 57.07; H, 3.98; N, 7.78; S, 8.93.

#### 3.1.8. 6-Ethyl-4-oxo-N-(4-sulfamoylphenyl)-4*H*-chromene-2-carboxamide (**5d**)

84% yield. MP = 297–299 °C. ^1^H NMR (400 MHz, DMSO-*d*_6_) *δ*: 10.92 (s, 1H, NH), 7.98 (d, *J* = 6.9 Hz, 2H, Ar), 7.87–7.83 (m, 4H, Ar), 7.74 (d, *J* = 1.5 Hz, 1H, Ar), 7.25 (s, 2H, SO_2_NH_2_), 6.97 (s, 1H, Ar), 2.78 (q, *J* = 7.6 Hz, 2H, CH_2_CH_3_), 1.29 (t, *J* = 7.6 Hz, 3H, CH_2_CH_3_). ^13^C NMR (151 MHz, DMSO-*d*_6_) δ: 177.68, 158.66, 155.55, 154.00, 142.43, 140.94, 140.41, 135.58, 127.09, 123.96, 123.36, 121.19, 119.34, 111.65, 40.51, 27.98, 15.79. MS (ESI): *m*/*z* (%) = 373.1 (21.4) [M + H]^+^. Anal. Calcd. for C_18_H_16_N_2_O_5_S (%): C, 58.05; H, 4.33; N, 7.52; S, 8.61. Found (%): C, 58.07; H, 4.37; N, 7.58; S, 8.63.

#### 3.1.9. 6,7-Dimethyl-4-oxo-N-(4-sulfamoylphenyl)-4*H*-chromene-2-carboxamide (**5e**)

87% yield. MP = 310–312 °C. ^1^H NMR (400 MHz, DMSO-*d*_6_) *δ*: 10.95 (s, 1H, NH), 7.98 (d, *J* = 8.7 Hz, 2H, Ar), 7.87 (d, *J* = 8.7 Hz, 2H, Ar), 7.80 (s, 1H, Ar), 7.60 (s, 1H, Ar), 7.35 (s, 2H, SO_2_NH_2_), 6.93 (s, 1H, Ar), 2.41 (s, 3H, CH_3_), 2.34 (s, 3H, CH_3_). MS (ESI): *m*/*z* (%) = 373.1 (21.4) [M + H]^+^. Anal. Calcd. for C_18_H_16_N_2_O_5_S (%): C, 58.05; H, 4.33; N, 7.52; S, 8.61. Found (%): C, 58.03; H, 4.32; N, 7.51; S, 8.65.

#### 3.1.10. 7,8-Dimethyl-4-oxo-N-(4-sulfamoylphenyl)-4*H*-chromene-2-carboxamide (**5f**)

90% yield. MP = 328–330 °C. ^1^H NMR (400 MHz, DMSO-*d*_6_) *δ*: 10.85 (s, 1H, NH), 7.96 (d, *J* = 8.7 Hz, 2H, Ar), 7.87 (d, *J* = 8.7 Hz, 2H, Ar), 7.81 (d, *J* = 8.1 Hz, 1H, Ar), 7.38–7.36 (m, 3H, (1H, Ar + SO_2_NH_2_), 7.00 (s, 1H, Ar), 2.48 (s, 3H, CH_3_), 2.43 (s, 3H, CH_3_). ^13^C NMR (151 MHz, DMSO-*d*_6_) δ: 177.86, 162.72, 159.18, 155.74, 153.92, 144.78, 141.02, 140.36, 128.04, 127.10, 126.54, 122.18, 122.02, 121.02, 111.44, 40.86, 40.48, 36.22, 31.21, 20.51, 12.04. MS (ESI): *m*/*z* (%) = 373.1 (21.2) [M + H]^+^. Anal. Calcd. for C_18_H_16_N_2_O_5_S (%): C, 58.05; H, 4.33; N, 7.52; S, 8.61. Found (%): C, 58.09; H, 4.35; N, 7.48; S, 8.67.

#### 3.1.11. 5,7-Dimethyl-4-oxo-N-(4-sulfamoylphenyl)-4*H*-chromene-2-carboxamide (**5g**)

76% yield. MP = 304–306 °C. ^1^H NMR (400 MHz, DMSO-*d*_6_) *δ*: 10.86 (s, 1H, NH), 7.98 (d, *J* = 8.7 Hz, 2H, Ar), 7.87 (d, *J* = 8.7 Hz, 2H, Ar), 7.46 (s, 1H, Ar), 7.31 (s, 2H, SO_2_NH_2_), 7.12 (s, 1H, Ar), 6.85 (s, 1H, Ar), 2.73 (s, 3H, CH_3_), 2.44 (s, 3H, CH_3_). ^13^C NMR (126 MHz, DMSO-*d*_6_) δ: 179.43, 158.61, 157.08, 153.85, 145.14, 140.96, 140.35, 139.94, 130.04, 127.08, 121.12, 120.26, 116.97, 113.00, 22.42, 21.54. MS (ESI): *m*/*z* (%) = 373.1 (21.4) [M + H]^+^. Anal. Calcd. for C_18_H_16_N_2_O_5_S (%): C, 58.05; H, 4.33; N, 7.52; S, 8.61. Found (%): C, 58.07; H, 4.37; N, 7.56; S, 8.64.

#### 3.1.12. 6,8-Dimethyl-4-oxo-N-(4-sulfamoylphenyl)-4*H*-chromene-2-carboxamide (**5h**)

88% yield. MP = 309–311 °C. ^1^H NMR (400 MHz, DMSO-*d*_6_) *δ*: 10.87 (s, 1H, NH), 7.93 (d, *J* = 8.7 Hz, 2H, Ar), 7.88 (d, *J* = 8.7 Hz, 2H, Ar), 7.48 (s, 1H, Ar), 7.42 (s, 2H, SO_2_NH_2_), 7.23 (s, 1H, Ar), 6.87 (s, 1H, Ar), 2.63 (s, 3H, CH_3_), 2.42 (s, 3H, CH_3_). MS (ESI): *m*/*z* (%) = 373.1 (21.5) [M + H]^+^. Anal. Calcd. for C_18_H_16_N_2_O_5_S (%): C, 58.05; H, 4.33; N, 7.52; S, 8.61. Found (%): C, 58.11; H, 4.30; N, 7.51; S, 8.70.

#### 3.1.13. 6-Chloro-4-oxo-N-(4-sulfamoylphenyl)-4*H*-chromene-2-carboxamide (**5i**)

92% yield. MP = 296–298 °C. ^1^H NMR (400 MHz, DMSO-*d*_6_) *δ*: 10.92 (s, 1H, NH), 8.00–7.84 (m, 7H, Ar), 7.23 (s, 2H, SO_2_NH_2_), 7.02 (s, 1H, Ar). ^13^C NMR (151 MHz, DMSO-*d*_6_) δ: 176.66, 158.30, 155.88, 154.12, 140.84, 140.49, 135.47, 131.09, 127.12, 125.26, 124.40, 121.90, 121.21, 111.72, 40.51. MS (ESI): *m*/*z* (%) = 380.0 (39.2) [M + H]^+^. Anal. Calcd. for C_16_H_11_ClN_2_O_5_S (%): C, 50.73; H, 2.93; Cl, 9.36; N, 7.40; S, 8.47. Found (%): C, 50.81; H, 2.95; Cl, 9.38; N, 7.45; S, 8.53.

#### 3.1.14. 6-Chloro-7-methyl-4-oxo-N-(4-sulfamoylphenyl)-4*H*-chromene-2-carboxamide (**5j**)

92% yield. MP = 305–307 °C. ^1^H NMR (400 MHz, DMSO-*d*_6_) *δ*: 10.97 (s, 1H, NH), 8.00–7.85 (m, 6H, Ar), 7.35 (s, 2H, SO_2_NH_2_), 7.00 (s, 1H, Ar), 2.52 (s, 3H, CH_3_). ^13^C NMR (151 MHz, DMSO-*d*_6_) δ: 176.46, 158.38, 155.69, 153.94, 144.05, 140.89, 140.45, 131.91, 127.13, 124.64, 123.35, 121.53, 121.11, 111.66, 40.51, 20.75. MS (ESI): *m*/*z* (%) = 394.0 (39.5) [M + H]^+^. Anal. Calcd. for C_17_H_13_ClN_2_O_5_S (%): C, 51.98; H, 3.34; Cl, 9.03; N, 7.13; S, 8.16. Found (%): C, 52.03; H, 3.27; Cl, 9.08; N, 7.15; S, 8.21.

#### 3.1.15. General Procedure for Synthesis of Compounds **6a**–**k**

The corresponding 4-oxo-4*H*-chromene-2-carboxylic acid (**2a**–**j**) (1.52 mmol, 1 eq.) was dissolved in DMF (10 mL), and 1,1-carbonyldiimidazole (0.320 g, 1.98 mmol, 1.3 eq.) was added in one portion. The reaction mixture was stirred at 80–90 °C for 1.2 h; afterward, the corresponding amine (**4a**,**b**) (1.82 mmol, 1.2 eq.) in DMF (7 mL) was added and the mixture was continually heated for another 1 h. After cooling to room temperature, the reaction mixture was treated with water (20 mL) and extracted with ethyl acetate (2 × 30 mL), washed with brine, and dried with Na_2_SO_4_. The solvent was evaporated under reduced pressure and the residue was purified by HPLC to afford the desired amide (**6a**–**k**).

#### 3.1.16. 4-Oxo-N-(4-sulfamoylbenzyl)-4*H*-chromene-2-carboxamide (**6a**)

96% yield. MP = 284–286 °C. ^1^H NMR (400 MHz, CF_3_COOD) *δ*: 8.31 (d, *J* = 6.7 Hz, 1H, Ar), 8.08–7.95 (m, 3H, Ar), 7.77 (m, 4H, Ar), 7.56 (s, 1H, Ar), 4.88 (s, 2H, CH_2_). ^13^C NMR (126 MHz, DMSO-*d*_6_) δ: 177.74, 159.47, 156.02, 155.51, 143.71, 142.64, 135.46, 129.60, 126.47, 126.23, 125.39, 124.07, 119.19, 110.89, 40.90, 34.86. MS (ESI): *m*/*z* (%) = 359.1 (20.3) [M + H]^+^. Anal. Calcd. for C_17_H_14_N_2_O_5_S (%): C, 56.98; H, 3.94; N, 7.82; S, 8.95. Found (%): C, 57.02; H, 3.87; N, 7.79; S, 8.94.

#### 3.1.17. 6-Methyl-4-oxo-N-(4-sulfamoylbenzyl)-4*H*-chromene-2-carboxamide (**6b**)

91% yield. MP = 293–295 °C. ^1^H NMR (400 MHz, DMSO-*d*_6_) *δ*: 9.75 (t, *J* = 6.0 Hz, 1H, NH), 7.84 (d, *J* = 2.1 Hz, 1H, Ar), 7.83–7.78 (m, 2H, Ar), 7.70 (dd, *J* = 8.6, 2.3 Hz, 1H, Ar), 7.61 (d, *J* = 8.6 Hz, 1H, Ar), 7.57–7.50 (m, 2H, Ar), 7.34 (s, 2H, SO_2_NH_2_), 6.84 (d, *J* = 1.5 Hz, 1H, Ar), 4.58 (d, *J* = 6.0 Hz, 2H, CH_2_), 2.43 (s, 3H, CH_3_). ^13^C NMR (151 MHz, DMSO-*d*_6_) δ: 177.67, 159.81, 155.75, 153.81, 143.34, 142.97, 136.50, 136.15, 128.22, 126.23, 124.63, 123.83, 118.99, 110.97, 42.83, 40.51, 20.91. MS (ESI): *m*/*z* (%) = 373.1 (21.5) [M + H]^+^. Anal. Calcd. for C_18_H_16_N_2_O_5_S (%): C, 58.05; H, 4.33; N, 7.52; S, 8.61. Found (%): C, 58.10; H, 4.29; N, 7.50; S, 8.71.

#### 3.1.18. 6-Chloro-4-oxo-N-(4-sulfamoylbenzyl)-4*H*-chromene-2-carboxamide (**6c**)

84% yield. MP = 299–301 °C. ^1^H NMR (400 MHz, DMSO-*d*_6_) *δ*: 9.74 (s, 1H, NH), 8.01–7.89 (m, 2H, Ar), 7.78 (ddd, *J* = 18.4, 8.6, 1.8 Hz, 3H, Ar), 7.53 (d, *J* = 7.9 Hz, 2H, Ar), 7.29 (s, 2H, SO_2_NH_2_), 6.89 (d, *J* = 1.7 Hz, 1H, Ar), 4.59 (d, *J* = 6.0 Hz, 2H, CH_2_). ^13^C NMR (126 MHz, DMSO-*d*_6_) δ: 176.72, 159.49, 156.09, 154.12, 143.36, 142.87, 135.38, 130.91, 128.23, 126.23, 125.23, 124.39, 121.69, 111.05, 42.83. MS (ESI): *m*/*z* (%) = 394.0 (39.5) [M + H]^+^. Anal. Calcd. for C_17_H_13_ClN_2_O_5_S (%): C, 51.98; H, 3.34; Cl, 9.03; N, 7.13; S, 8.16. Found (%): C, 52.04; H, 3.28; Cl, 9.09; N, 7.16; S, 8.22.

#### 3.1.19. 4-Oxo-N-(4-sulfamoylphenethyl)-4*H*-chromene-2-carboxamide (**6d**)

95% yield. MP = 248–250 °C. ^1^H NMR (400 MHz, DMSO-*d*_6_) *δ*: 9.24–9.17 (m, 1H, NH), 8.05 (dd, *J* = 8.0, 1.5 Hz, 1H, Ar), 7.89 (ddd, *J* = 8.5, 7.1, 1.7 Hz, 1H, Ar), 7.77 (d, *J* = 7.9 Hz, 2H, Ar), 7.71 (d, *J* = 8.5 Hz, 1H, Ar), 7.53 (t, *J* = 7.6 Hz, 1H, Ar), 7.46 (d, *J* = 8.0 Hz, 2H), 7.27 (s, 2H, SO_2_NH_2_), 6.82 (s, 1H, Ar), 3.58 (q, *J* = 6.9 Hz, 2H, NCH_2_), 2.98 (t, *J* = 7.4 Hz, 2H, CH_2_Ar). MS (ESI): *m*/*z* (%) = 373.1 (21.5) [M + H]^+^. Anal. Calcd. for C_18_H_16_N_2_O_5_S (%): C, 58.05; H, 4.33; N, 7.52; S, 8.61. Found (%): C, 58.10; H, 4.29; N, 7.50; S, 8.71.

#### 3.1.20. 6-Methyl-4-oxo-N-(4-sulfamoylphenethyl)-4*H*-chromene-2-carboxamide (**6e**)

84% yield. MP = 257–259 °C. ^1^H NMR (400 MHz, DMSO-*d*_6_) *δ*: 9.18–9.14 (m, 1H, NH), 7.82 (s, 1H, Ar), 7.89 (d, *J* = 8.5 Hz, 2H, Ar), 7.77–7.61 (m, 2H, Ar), 7.42 (d, *J* = 8.5 Hz, 2H, Ar), 7.19 (s, 2H, SO_2_NH_2_), 6.77 (s, 1H, Ar), 3.54 (q, *J* = 6.9 Hz, 2H, NCH_2_), 2.96 (t, *J* = 7.4 Hz, 2H, CH_2_Ar), 2.46 (s, 3H, CH_3_). MS (ESI): *m*/*z* (%) = 387.1 (22.5) [M + H]^+^. Anal. Calcd. for C_19_H_18_N_2_O_5_S (%): C, 59.06; H, 4.70; N, 7.25; S, 8.30. Found (%): C, 59.10; H, 4.79; N, 7.23; S, 8.33.

#### 3.1.21. 7-Methyl-4-oxo-N-(4-sulfamoylphenethyl)-4*H*-chromene-2-carboxamide (**6f**)

87% yield. MP = 253–255 °C. ^1^H NMR (400 MHz, DMSO-*d*_6_) *δ*: 9.23 (t, *J* = 6.9 Hz, 1H, NH), 7.92 (d, *J* = 8.4 Hz, 1H, Ar), 7.75 (d, *J* = 8.0 Hz, 2H, Ar), 7.50 (s, 1H, Ar), 7.44 (d, *J* = 8.0 Hz, 2H, Ar), 7.35 (d, *J* = 8.4 Hz, 2H, Ar), 7.30 (s, 2H, SO_2_NH_2_), 6.76 (s, 1H, Ar), 3.55 (q, *J* = 6.9 Hz, 2H, NCH_2_), 2.96 (t, *J* = 7.4 Hz, 2H, CH_2_Ar), 2.47 (s, 3H, CH_3_). MS (ESI): *m*/*z* (%) = 387.1 (22.5) [M + H]^+^. Anal. Calcd. for C_19_H_18_N_2_O_5_S (%): C, 59.06; H, 4.70; N, 7.25; S, 8.30. Found (%): C, 59.12; H, 4.73; N, 7.24; S, 8.35.

#### 3.1.22. 6-Ethyl-4-oxo-N-(4-sulfamoylphenethyl)-4*H*-chromene-2-carboxamide (**6g**)

90% yield. MP = 257–259 °C. ^1^H NMR (400 MHz, DMSO-*d*_6_) *δ*: 9.21 (s, 1H, NH), 7.85 (d, *J* = 2.2 Hz, 1H, Ar), 7.80–7.71 (m, 3H, Ar), 7.63 (d, *J* = 8.6 Hz, 1H, Ar), 7.46 (d, *J* = 8.3 Hz, 2H, Ar), 7.28 (s, 2H, SO_2_NH_2_), 6.79 (s, 1H, Ar), 3.56 (q, *J* = 6.8 Hz, 2H, NCH_2_), 2.97 (t, *J* = 6.9 Hz, 2H, CH_2_Ar), 2.75 (q, *J* = 7.6 Hz, 2H, CH_2_Ar), 1.23 (t, *J* = 7.6 Hz, 3H, CH_3_). ^13^C NMR (126 MHz, DMSO-*d*_6_) δ: 177.72, 159.53, 155.90, 153.94, 143.71, 142.64, 142.27, 135.48, 129.59, 126.22, 123.87, 123.37, 119.10, 110.72, 40.89, 34.86, 27.97, 15.83. MS (ESI): *m*/*z* (%) = 387.1 (22.5) [M + H]^+^. Anal. Calcd. for C_20_H_20_N_2_O_5_S (%): C, 59.99; H, 5.03; N, 7.00; S, 8.01. Found (%): C, 59.96; H, 5.07; N, 7.04; S, 8.05.

#### 3.1.23. 6,7-Dimethyl-4-oxo-N-(4-sulfamoylphenethyl)-4*H*-chromene-2-carboxamide (**6h**)

78% yield. MP = 282–284 °C. ^1^H NMR (400 MHz, DMSO-*d*_6_) *δ*: 9.20 (t, *J* = 5.7 Hz, 1H, NH), 7.76 (dd, *J* = 6.3, 1.9 Hz, 3H, Ar), 7.46 (d, *J* = 8.2 Hz, 3H, Ar), 7.31 (s, 2H, SO_2_NH_2_), 6.74 (d, *J* = 1.2 Hz, 1H, Ar), 3.56 (q, *J* = 6.8 Hz, 2H, NCH_2_), 2.96 (t, *J* = 7.3 Hz, 2H, CH_2_Ar), 2.38 (s, 3H, CH_3_), 2.32 (s, 3H, CH_3_). ^13^C NMR (151 MHz, DMSO-*d*_6_) δ: 177.42, 159.61, 155.65, 153.99, 145.63, 143.71, 142.64, 135.60, 129.58, 126.22, 124.84, 121.97, 118.95, 110.74, 40.85, 40.51, 34.85, 20.47, 19.36. MS (ESI): *m*/*z* (%) = 387.1 (22.5) [M + H]^+^. Anal. Calcd. for C_20_H_20_N_2_O_5_S (%): C, 59.99; H, 5.03; N, 7.00; S, 8.01. Found (%): C, 59.94; H, 5.05; N, 7.02; S, 8.03.

#### 3.1.24. 7,8-Dimethyl-4-oxo-N-(4-sulfamoylphenethyl)-4*H*-chromene-2-carboxamide (**6i**)

82% yield. MP = 280–282 °C. ^1^H NMR (400 MHz, DMSO-*d*_6_) *δ*: 8.75 (t, *J* = 6.0 Hz, 1H, NH), 7.77–7.74 (m, 3H, Ar), 7.43 (d, *J* = 8.1 Hz, 2H, Ar), 7.30 (d, *J* = 8.4 Hz, 1H, Ar), 7.20 (s, 2H, SO_2_NH_2_), 6.75 (d, *J* = 1.2 Hz, 1H, Ar), 3.60 (q, *J* = 6.0 Hz, 2H, NCH_2_), 2.96 (t, *J* = 7.3 Hz, 2H, CH_2_Ar), 2.52 (s, 3H, CH_3_), 2.48 (s, 3H, CH_3_). MS (ESI): *m*/*z* (%) = 387.1 (22.5) [M + H]^+^. Anal. Calcd. for C_20_H_20_N_2_O_5_S (%): C, 59.99; H, 5.03; N, 7.00; S, 8.01. Found (%): C, 59.91; H, 5.08; N, 7.05; S, 8.00.

#### 3.1.25. 6,8-Dimethyl-4-oxo-N-(4-sulfamoylphenethyl)-4*H*-chromene-2-carboxamide (**6j**)

80% yield. MP = 283–285 °C. ^1^H NMR (400 MHz, DMSO-*d*_6_) *δ*: 8.81 (t, *J* = 6.8 Hz, 1H, NH), 7.77 (d, *J* = 8.0 Hz, 2H, Ar), 7.65 (s, 1H, Ar), 7.53 (s, 1H, Ar), 7.47 (d, *J* = 8.0 Hz, 2H, Ar), 7.27 (s, 2H, SO_2_NH_2_), 6.76 (s, 1H, Ar), 3.58 (q, *J* = 6.8 Hz, 2H, NCH_2_), 2.97 (t, *J* = 7.2 Hz, 2H, CH_2_Ar), 2.49 (s, 3H, CH_3_), 2.38 (s, 3H, CH_3_). ^13^C NMR (126 MHz, DMSO-*d*_6_) δ: 177.84, 159.87, 156.03, 152.33, 143.72, 142.66, 137.18, 135.53, 129.64, 128.33, 126.21, 123.82, 122.17, 110.72, 40.94, 34.85, 20.85, 15.73. MS (ESI): *m*/*z* (%) = 387.1 (22.5) [M + H]^+^. Anal. Calcd. for C_20_H_20_N_2_O_5_S (%): C, 59.99; H, 5.03; N, 7.00; S, 8.01. Found (%): C, 60.03; H, 5.04; N, 7.02; S, 8.06.

#### 3.1.26. 6-Chloro-4-oxo-N-(4-sulfamoylphenethyl)-4*H*-chromene-2-carboxamide (**6k**)

73% yield. MP = 285–287 °C. ^1^H NMR (400 MHz, DMSO-*d*_6_) *δ*: 9.26 (t, *J* = 5.6 Hz, 1H, NH), 7.99–7.88 (m, 2H, Ar), 7.75 (t, *J* = 8.9 Hz, 3H, Ar), 7.46 (d, *J* = 8.1 Hz, 2H, Ar), 7.29 (s, 2H, SO_2_NH_2_), 6.83 (s, 1H, Ar), 3.62–3.51 (m, 2H, NCH_2_), 2.96 (t, *J* = 7.3 Hz, 2H, CH_2_Ar). ^13^C NMR (126 MHz, DMSO-*d*_6_) δ: 176.70, 159.21, 156.20, 154.08, 143.66, 142.65, 135.35, 130.90, 129.59, 126.23, 125.19, 124.38, 121.65, 110.81, 40.91, 34.84. MS (ESI): *m*/*z* (%) = 408.0 (39.7) [M + H]^+^. Anal. Calcd. for C_18_H_15_ClN_2_O_5_S (%): C, 53.14; H, 3.72; Cl, 8.71; N, 6.89; S, 7.88. Found (%): C, 53.04; H, 3.78; Cl, 8.79; N, 7.16; S, 7.82.

### 3.2. Carbonic Anhydrase Inhibition

An Applied Photophysics stopped-flow instrument was used for assaying the CA catalyzed CO_2_ hydration activity [38]. Phenol red (at a concentration of 0.2 mM) was used as an indicator, working at the absorbance maximum of 557 nm, with 20 mM Hepes (pH 7.4) as a buffer, and 20 mM Na_2_SO_4_ (for maintaining constant ionic strength), following the initial rates of the CA-catalyzed CO_2_ hydration reaction for a period of 10–100 s. The CO_2_ concentrations ranged from 1.7 to 17 mM for the determination of the kinetic parameters and inhibition constants [39]. The non-catalyzed CO_2_ hydration was not subtracted from these curves and accounts for the remaining observed activity even at a high concentration of an inhibitor, being in the range of 16–25%. However, the background activity from the uncatalyzed reaction is always subtracted when IC_50_ values are obtained by using the data analysis software for the stopped flow instrument. Enzyme concentrations ranged between 5–12 nM. For each inhibitor, at least six traces of the initial 5–10% of the reaction were used for determining the initial velocity. The uncatalyzed rates were determined in the same manner and subtracted from the total observed rates. Stock solutions of the inhibitor (0.1 mM) were prepared in distilled, deionized water, and dilutions up to 0.01 nM were done thereafter with the assay buffer. Inhibitor and enzyme solutions were preincubated together for 15 min at room temperature prior to the assay, allowing the formation of the E–I complex. The inhibition constants were obtained by non-linear least-squares methods using PRISM 3 and the Cheng-Prusoff equation as reported earlier, which represent the mean from at least three different determinations. All CA isoforms were recombinant proteins obtained in house, as reported earlier [40,41,42].

### 3.3. Molecular Modeling Studies (Experimental Part)

Docking calculations were carried out using the AutoDock 4.2 software [43]. The free energy of binding (DG) of the cytosolic isoforms hCA I and II as well as the transmembrane tumor-associated ones IX and XII in complex with the tested compounds was generated using this molecular docking program. The crystal structures of hCA I (PDB code 3W6H), hCA II (PDB code 3HS4), hCA IX (PDB code 3IAI), and hCA XII (PDB code 1JD0) were obtained from the Protein Data Bank [44]. For the enzymes’ preparation, all water molecules were eliminated polar hydrogens, which were added, and the co-crystallized ligands were removed from each enzyme’s active site, while for the preparation of the inhibitors, the charges were added and the rotatable bonds determined. Grid maps have been calculated utilizing Autogrid algorithm and must contain the area to be connected. A set of grids of 60 Å × 50 Å × 50 Å with 0.375 Å spacing was calculated, considering the docking area for all the ligand atom types employing AutoGrid4. Three-dimensional structures of all compounds were constructed using Chem3Dultra 12.0 software (Chemical Structure Drawing Standard; Perkin Elmer Informatics, Waltham, MA, USA). For the present system, the Lamarckian genetic algorithm was applied for minimization using default parameters. The pitch was 1.0 Å, while the quaternion and pivot angle were set to 5 and 0 degrees. For each compound, 200 configurations were produced. The results from the Autodock calculations were grouped using a root mean standard deviation (RMSD) value of 1.5 Å, while the lowest-energy configuration of the largest population group was chosen as the most likely tethering configuration. The LigandScout software program (inte:ligand, Vienna, Austria) was used to display the results and process of the configurations with the highest tie rating. Finally, the docking protocol was verified by re-docking of the co-crystallized ligand acetazolamide (AAZ) in the vicinity of the active sites of each enzyme with RMSD values 0.885, 0.966, 1.034, and 1.176 Å for hCA I, II, IX, and XII, respectively.

## 4. Conclusions

In conclusion, we synthetized and investigated two novel series of chromene-containing aromatic sulfonamides for their effective inhibition against different and most relevant human carbonic anhydrase isoforms such as the ubiquitous hCA I, hCA II, and the tumor associate isoforms hCA IX and XII, which are involved in a variety of diseases such as glaucoma, retinitis pigmentosa, epilepsy, and tumors. Compound **5a** showed a good selectivity index of 31.67 on hCA IX compared to hCA I and over two times compared to hCA II. On the other hand, compound **6f** showed to be the most active hCA II with an SI of 44.47 compared to hCA I and 44.97 compared to hCA XII. In addition, the methylene linker among the chromene scaffold and sulfonamide moiety played a crucial role in the modulation of potency and selectivity inhibition against different isoforms. Indeed, compound **5b** showed a Ki of 77 nM against hCA IX and the analog **6b**, with a methylene linker, and dramatically decreased the potency to 2039 nM, losing the selective inhibition against this isoform. These interesting features make them good candidates for preclinical evaluation in glaucoma or various tumors in which the two enzymes (hCA II and hCA IX) are involved. Furthermore, computational procedures were used to investigate the binding mode of this class of compounds within the active site of hCA IX.

## Figures and Tables

**Table 1:** Inhibition data of human CA isoforms hCA I, II, IX, and XII with compounds 1–14 and the standard sulfonamide inhibitor acetazolamide (AAZ).

K_i_ (nM) *					K_i_ (nM) *				
N	hCA I	hCA II	hCA IX	hCA XII	N	hCA I	hCA II	hCA IX	hCA XII
**5a**	525.2 ± 44.4	35.1 ± 3.3	16.6 ± 1.5	20.1 ± 1.7	**6b**	612.3 ± 49.5	81.7 ± 4.2	2039 ± 189.0	320.4 ± 23.2
**5b**	5314 ± 272	95.9 ± 8.0	77 ± 6.5	64.8 ± 6.1	**6c**	2646 ± 227	678.8 ± 45.0	3285 ± 302	59.4 ± 5.0
**5c**	3563 ± 228	39.5 ± 3.2	63.4 ± 6.3	50.8 ± 4.3	**6d**	321.9 ± 27.9	16.6 ± 1.2	81.0 ± 7.7	211.6 ± 13.7
**5e**	2891 ± 161	311.4 ± 28.3	298.6 ± 18.4	619.1 ± 36.7	**6e**	246.7 ± 24.3	36.4 ± 2.1	72.7 ± 4.3	439.3 ± 32.2
**5f**	341.1 ± 29.6	9.3 ± 0.5	19.5 ± 1.6	76.3 ± 6.0	**6f**	333.5 ± 17.5	7.5 ± 0.5	64.8 ± 3.4	337.3 ± 27.4
**5g**	681.5 ± 40.6	61.4 ± 4.4	54.7 ± 5.0	46.4 ± 4.4	**6g**	971.0 ± 82.4	279.9 ± 15.5	182.6 ± 16.1	147.9 ± 8.1
**5h**	504.8 ± 27.0	46.6 ± 2.9	22.5 ± 1.9	26.8 ± 1.4	**6h**	547.3 ± 39.2	51.2 ± 3.6	51.4 ± 4.5	22.6 ± 1.5
**5i**	2698 ± 265	66.2 ± 5.8	51.0 ± 4.8	424.7 ± 41.0	**6i**	213.6 ± 15.9	39.2 ± 3.7	84.5 ± 4.3	132.2 ± 11.0
**5j**	580.3 ± 50.5	900.7 ± 75.4	515.5 ± 37.2	155.3 ± 10.1	**6j**	4412 ± 347	143.0 ± 12.8	91.1 ± 8.9	31.4 ± 2.6
**6a**	627.7 ± 35.5	219.3 ± 11.8	855.2 ± 49.2	34.0 ± 2.2	**6k**	926.9 ± 68.9	91.6 ± 7.0	1917 ± 146	41.4 ± 3.4
**AAZ**	250 ± 13.0	12.1 ± 0.6	25.7 ± 2.1	5.7 ± 0.4	**AAZ**	250 ± 13.0	12.1 ± 0.6	25.7 ± 2.1	5.7 ± 0.4

* Mean from three different assays, by a stopped flow technique (errors were in the range of 5–10% of the reported values).

**Table 2:** Molecular docking free binding energies (kcal/mol) and interactions of tested compounds on hCA I, II, IX, and XII isoforms.

No.	hCA Isoform	Estimated Free Binding Energy (Kcal/mol)	Chelating the Zn(II) Ion	Residues Involved in H-Bond Interactions	Residues Involved in Hydrophobic Interactions
**5a**	hCA I	−6.49	Yes	Thr199	Ala121, Leu198
	hCA II	−7.42	Yes	Thr199	Ile91, Val121, Leu198, Thr200
	hCA IX	−9.25	Yes	His94, Thr199	Val121, Val195, Leu198
	hCA XII	−9.61	Yes	His94, His96, Thr199, Thr200	Val121, Leu198
**5h**	hCA I	−5.17	No	-	Ala121, Ala135
	hCA II	−7.11	Yes	Thr199	Ile91, Leu198, Thr200
	hCA IX	−8.74	Yes	His94, Thr199	Val121, Leu198
	hCA XII	−9.02	Yes	His94, Thr200	Val121, Leu198
**6d**	hCA I	−6.77	Yes	Thr199	Ala121, Ala135, Leu198
	hCA II	−9.14	Yes	His94, Thr199	Ile91, Val121, Phe131, Thr200
	hCA IX	−6.44	Yes	Thr199	Val121, Leu198, Thr200
	hCA XII	−4.81	No	-	Val121
**6f**	hCA I	−6.62	Yes	Thr199	Ala135, Leu198
	hCA II	−9.34	Yes	His94, Thr199	Ile91, Val121, Phe131, Leu198, Thr200
	hCA IX	−6.81	Yes	Thr199	Val121, Leu198, Thr200
	hCA XII	−4.72	No	-	Val121, Thr200
**6h**	hCA I	−5.29	Yes	-	Ala135, Leu198
	hCA II	−7.87	Yes	His94	Val121, Phe131, Leu198
	hCA IX	−6.53	Yes	Thr199	Val121, Thr200
	hCA XII	−9.52	Yes	Ala131, Ser132, Thr200	Trp5, Val121, Leu198
**6i**	hCA I	−8.15	Yes	Gln92, Thr199	Leu131, Ala135, Ala132, Thr202, Leu198
	hCA II	−7.14	Yes	Thr200	Val121, Phe131, Thr200
	hCA IX	−6.27	Yes	Thr199	Val121, Thr200
	hCA XII	−4.35	No	-	Val121, Leu198
**AAZ**	hCA I		Yes	Gln92	Leu198, Thr199, His200, Pro 201, Trp209
	hCA II		Yes	Thr199, Thr200	Val121, Phe131, Leu198, Trp209
	hCA IX		Yes	Thr199, Thr200	Val121, Val143, Val131, Leu198, Trp209
	hCA XII		Yes	Thr199, Thr200	Val121, Val143, Leu198, Trp209

- Means that there is no residues involved in H –bond formation.

## Data Availability

we have no supporting information.

## Abbreviations

CA	Carbonic Anhydrase
CAI	Carbonic Anhydrase Inhibitors
SAR	Structure Activity Relationships
SI	Selectivity Index

## References

[1] Neri D., Supuran C.T. (2011). Interfering with pH regulation in tumours as a therapeutic strategy. Nat. Rev. Drug Discov..

[2] Supuran C.T., Scozzafava A. (2007). Carbonic anhydrases as targets for medicinal chemistry. Bioorg. Med. Chem..

[3] Supuran C.T. (2008). Carbonic anhydrases: Novel therapeutic applications for inhibitors and activators. Nat. Rev. Drug Discov..

[4] Supuran C.T. (2010). Carbonic anhydrase inhibitors. Bioorg. Med. Chem. Lett..

[5] Supuran C.T. (2011). Carbonic anhydrase inhibitors and activators for novel therapeutic applications. Future Med. Chem..

[6] Alterio V., Di Fiore A., D’Ambrosio K., Supuran C.T., De Simone G. (2012). Multiple binding modes of inhibitors to carbonic an-hydrases: How to design specific drugs targeting 15 different isoforms?. Chem. Rev..

[7] Thiry A., Dogné J.-M., Masereel B., Supuran C.T. (2006). Targeting tumor-associated carbonic anhydrase IX in cancer therapy. Trends Pharmacol. Sci..

[8] Tuccinardi T., Ortore G., Rossello A., Supuran A.C.T., Martinelli A. (2007). Homology Modeling and Receptor-Based 3D-QSAR Study of Carbonic Anhydrase IX. J. Chem. Inf. Model..

[9] Vullo D., Innocenti A., Nishimori I., Pastorek J., Scozzafava A., Pastorekova S., Supuran C.T. (2005). Carbonic anhydrase inhibitors. Inhibition of the transmembrane isozyme XII with sulfonamides—A new target for the design of antitumor and an-tiglaucoma drugs?. Bioorg. Med. Chem. Lett..

[10] Masini E., Carta F., Scozzafava A., Supuran C.T. (2013). Antiglaucoma carbonic anhydrase inhibitors: A patent review. Expert Opin. Ther. Pat..

[11] Fouad S.A., Hessein S.A., Abbas S.Y., Farrag A.M., Ammar Y.A. (2018). Synthesis of Chromen-2-one, Pyrano[3,4-c]chromene and Pyridino[3,4-c]chromene Derivatives as Potent Antimicrobial Agents. Croat. Chem. Acta.

[12] Mashhadinezhad M., Mamaghani M., Rassa M., Shirini F. (2019). A Facile Green Synthesis of Chromene Derivatives as Antioxidant and Antibacterial Agents through a Modified Natural Soil. ChemistrySelect.

[13] Okasha R.M., Alblewi F.F., Afifi T.H., Naqvi A., Fouda A.M., Al-Dies A.M., El-Agrody A.M. (2017). Design of New Ben-zo[h]chromene Derivatives: Antitumor Activities and Structure-Activity Relationships of the 2,3-Positions and Fused Rings at the 2,3-Positions. Molecules.

[14] El-Agrody A.M., Fouda A.M., Assiri M.A., Mora A., Ali T.E., Alam M.M., Alfaifi M.Y. (2020). In vitro anticancer activity of py-rano[3, 2-c]chromene derivativeswith both cell cycle arrest and apoptosis induction. Med. Chem. Res..

[15] Alshibl H.M., Al-Abdullah E.S., Haiba M.E., Alkahtani H.M., Awad G.E., Mahmoud A.H., Ibrahim B.M., Bari A., Villinger A. (2020). Synthesis and Evaluation of New Coumarin Derivatives as Antioxidant, Antimicrobial, and Anti-Inflammatory Agents. Molecules.

[16] Chung S.-T., Huang W.-H., Huang C.-K., Liu F.-C., Huang R.-Y., Wu C.-C., Lee A.-R. (2016). Synthesis and anti-inflammatory activities of 4H-chromene and chromeno[2,3-b]pyridine derivatives. Res. Chem. Intermed..

[17] Awadallah F.M., El-Waei T.A., Hanna M.M., Abbas S.E., Ceruso M., Oz B.E., Guler O.O., Supuran C.T. (2015). Synthesis, carbonic anhydrase inhibition and cytotoxic activity of novel chromone-based sulfonamide derivatives. Eur. J. Med. Chem..

[18] Lomelino C.L., Supuran C.T., McKenna R. (2016). Non-Classical Inhibition of Carbonic Anhydrase. Int. J. Mol. Sci..

[19] Esirden I., Tanç M., Supuran C.T., Kaya M. (2017). Microwave assisted synthesis of novel tetrazole/sulfonamide derivatives based on octahydroacridine, xanthene and chromene skeletons as inhibitors of the carbonic anhydrases isoforms I, II, IV and VII. Bioorg. Med. Chem. Lett..

[20] Clima L., Craciun B.F., Angeli A., Petreni A., Bonardi A., Nocentini A., Carta F., Gratteri P., Pinteala M., Supuran C.T. (2020). Synthesis, Computational Studies and Assessment of in Vitro Activity of Squalene Derivatives as Carbonic Anhydrase Inhib-itors. ChemMedChem.

[21] Abbas H.S., Abd El-Karim S.S., Abdelwahed N. (2017). Synthesis and biological evaluation of sulfonamide derivatives as antimi-crobial agents. Acta Pol. Pharm..

[22] Azzam R.A., Elsayed R.E., Elgemeie G.H. (2020). Design, Synthesis, and Antimicrobial Evaluation of a New Series ofN Sulfonamide 2 Pyridones as Dual Inhibitors of DHPS and DHFR Enzymes. ACS Omega.

[23] Rakesh K.P., Wang S.-M., Leng J., Ravindar L., Asiri A.M., Marwani H.M., Qin H.-L. (2018). Recent Development of Sulfonyl or Sulfonamide Hybrids as Potential Anticancer Agents: A Key Review. Anticancer Agents Med. Chem..

[24] Kachaeva M.V., Hodyna D.M., Semenyuta I.V., Pilyo S.G., Prokopenko V.M., Kovalishyn V.V., Metelytsia L.O., Brovarets V.S. (2018). Design, synthesis and evaluation of novel sulfonamides as potential anticancer agents. Comput. Biol. Chem..

[25] Gao H.-D., Liu P., Yang Y., Gao F. (2016). Sulfonamide-1,3,5-triazine–thiazoles: Discovery of a novel class of antidiabetic agents via inhibition of DPP-4. RSC Adv..

[26] Irfan A., Rubab L., Rehman M.U., Anjum R., Ullah S., Marjana M., Qadeer S., Sana S. (2020). Coumarin sulfonamide derivatives: An emerging class of therapeutic agents. Heterocycl. Commun..

[27] Bhuva N.H., Talpara P.K., Singala P.M., Gothaliya V.K., Shah V.H. (2017). Synthesis and biological evaluation of pyrimidinyl sulphonamide derivatives as promising class of antitubercular agents. J. Saudi Chem. Soc..

[28] Cagide F., Oliveira C., Reis J., Borges F. (2019). Optimizing the Synthetic Route of Chromone-2-carboxylic Acids: A Step forward to Speed-Up the Discovery of Chromone-Based Multitarget-Directed Ligands. Molecules.

[29] Kanamori K., Roberts J.D. (1983). Nitrogen-15 nuclear magnetic resonance study of benzenesulfonamide and cyanate binding to carbonic anhydrase. Biochemistry.

[30] Alterio V., Hilvo M., Di Fiore A., Supuran C.T., Pan P., Parkkila S., Scaloni A., Pastorek J., Pastorekova S., Pedone C. (2009). Crystal structure of the catalytic domain of the tumor-associated human carbonic anhydrase IX. Proc. Natl. Acad. Sci. USA.

[31] Di Fiore A., Truppo E., Supuran C.T., Alterio V., Dathan N., Bootorabi F., Parkkila S., Monti S.M., De Simone G. (2010). Crystal structure of the C183S/C217S mutant of human CA VII in complex with acetazolamide. Bioorg. Med. Chem. Lett..

[32] Whittington D.A., Waheed A., Ulmasov B., Shah G.N., Grubb J.H., Sly W.S., Christianson D.W. (2001). Crystal structure of the dimeric extracellular domain of human carbonic anhydrase XII, a bitopic membrane protein overexpressed in certain cancer tumor cells. Proc. Natl. Acad. Sci. USA.

[33] Supuran C.T. (2012). Structure-based drug discovery of carbonic anhydrase inhibitors. J. Enzym. Inhib. Med. Chem..

[34] Wei L., Jiuhui L., Hongfeng S., Jiagao C., Li Z., Xu X. (2018). Synthesis, nematicidal activity and docking study of novel chromone derivatives containing substituted pyrazole. Chin. Chem. Lett..

[35] Sami S.M., Ibrahim S.S., Abdel-Halim A.M., Aly Y.L. (1986). Synthesis and Reactions of Some 4-Oxo-4*H*-1-benzopyran-2-carboxaldehydes. Indian J. Chem. Sec. B Org. Med. Chem..

[36] Helguera A.M., Pérez-Garrido A., Gaspar A., Reis J., Cagide F., Vina D., Cordeiro M.D., Borges F. (2013). Combining QSAR classification models for predictive modeling of human monoamine oxidase inhibitors. Eur. J. Med. Chem..

[37] Ellis G.P., Shaw D. (1972). Benzopyrones. 7. Synthesis and antiallergic activity of some 2-(5-tetrazolyl)chromones. J. Med. Chem..

[38] Khalifah R.G. (1971). The Carbon Dioxide Hydration Activity of Carbonic Anhydrase. J. Biol. Chem..

[39] Nocentini A., Angeli A., Carta F., Winum J.Y., Zalubovskis R., Carradori S., Capasso C., Donald W.A., Supuran C.T. (2021). Reconsidering anion inhibitors in the general context of drug design studies of modulators of activity of the classical enzyme carbonic anhydrase. J. Enzym. Inhib. Med. Chem..

[40] Bruno E., Buemi M.R., Di Fiore A., De Luca L., Ferro S., Angeli A., Cirilli R., Sadutto D., Alterio V., Monti S.M. (2017). Probing Molecular Interactions between Human Carbonic Anhydrases (hCAs) and a Novel Class of Benzenesulfonamides. J. Med. Chem..

[41] Angeli A., Carta F., Bartolucci G., Supuran C.T. (2017). Synthesis of novel acyl selenoureido benzensulfonamides as carbonic an-hydrase I, II, VII and IX inhibitors. Bioorg. Med. Chem..

[42] Vats L., Sharma V., Angeli A., Kumar R., Supuran C.T., Sharma P.K. (2018). Synthesis of novel 4-functionalized 1,5-diaryl-1,2,3-triazoles containing benzenesulfonamide moiety as carbonic anhydrase I, II, IV and IX inhibitors. Eur. J. Med. Chem..

[43] Morris G.M., Huey R., Lindstrom W., Sanner M.F., Belew R.K., Goodsell D.S. (2009). and Olson, A.J. Autodock4 and Auto-DockTools4: Automated docking with selective receptor flexiblity. J. Comput. Chem..

[44] RCSB, PDB A Structural View of Biology. http://www.rcsb.org/.

